# Anti-Inflammatory and Anti-Oxidant Effect of Dimethyl Fumarate in Cystic Fibrosis Bronchial Epithelial Cells

**DOI:** 10.3390/cells10082132

**Published:** 2021-08-19

**Authors:** Onofrio Laselva, Caterina Allegretta, Sante Di Gioia, Carlo Avolio, Massimo Conese

**Affiliations:** Department of Medical and Surgical Sciences, University of Foggia, 71122 Foggia, Italy; onofrio.laselva@unifg.it (O.L.); caterina.allegretta@unifg.it (C.A.); sante.digioia@unifg.it (S.D.G.); carlo.avolio@unifg.it (C.A.)

**Keywords:** dimethyl fumarate, cystic fibrosis, airway epithelial cells, cytokine, oxidative stress

## Abstract

Cystic Fibrosis (CF) is caused by mutations on the *CF transmembrane conductance regulator (CFTR)* gene and is associated with chronic infection and inflammation. Recently, it has been demonstrated that LPS-induced CFTR dysfunction in airway epithelial cells is due to an early oxidative stress. Dimethyl fumarate (DMF) is an approved anti-inflammatory and anti-oxidant drug for auto-immune and inflammatory diseases, but its role in the CF has never been investigated. In this study, we examined the effect of DMF on CF-related cytokines expression, ROS measurements and CFTR channel function. We found that DMF reduced the inflammatory response to LPS stimulation in both CF and non-CF bronchial epithelial cells, both as co-treatment and therapy, and restored LPS-mediated decrease of Trikafta™-mediated CFTR function in CF cells bearing the most common mutation, c.1521_1523delCTT (F508del). DMF also inhibited the inflammatory response induced by IL-1β/H_2_O_2_ and IL-1β/TNFα, mimicking the inflammatory status of CF patients. Finally, we also demonstrated that DMF exhibited an anti-oxidant effect on CF cells after different inflammatory stimulations. Since DMF is an approved drug, it could be further investigated as a novel anti-inflammatory molecule to ameliorate lung inflammation in CF and improve the CFTR modulators efficacy.

## 1. Introduction

Cystic fibrosis (CF) is the most common fatal autosomal recessive disorder among Caucasians, estimated to affect more than 70,000 people in the world. Over 2000 sequence variations have been discovered in the *CF Transmembrane Conductance Regulator (CFTR)* gene, ~300 of which are pathogenic [1], causing either lack or dysfunction of the mutated protein. The most common mutation is *F508del*, which accounts for approximately two thirds of all CFTR alleles in patients with CF. Lung disease represents the chief cause of morbidity and mortality of these patients, ultimately leading to chronic respiratory failure and lung transplantation. Its pathophysiology is characterized by severe and persistent bronchial inflammation and chronic bacterial infection, along with airway mucus obstruction and bronchiectasis. CF airways lack proper hydration of the mucus overlaying thexd epithelium, with consequent loss of the mucociliary clearance and opportunistic bacterial infections. Upon colonization by pathogens, the innate immunity of CF lungs prompts the secretion of pro-inflammatory cytokines (e.g., IL-1β, TNF-α) and chemokines (IL-8) by airway epithelial cells, macrophages and dendritic cells. These determine, at first, the attraction and activation of neutrophils, which, upon phagocytosis, kills pathogens [2]. However, for reasons still to be unveiled, this inflammatory response is not suited to combating and eradicating infections from the airways. Moreover, the acquired immunity is operated by T cell-mediated responses and, in particular, mediated by Th17 cells. CF lung secretions are enriched in IL-17A and IL-17F cytokines [3] which determine further activation of airway epithelial cells and stimulate lung fibroblast to secrete granulocyte macrophage colony stimulating factors (GM-CSF), IL-1β, IL-6 and TNF-α [4]. Each of these mediators produce an amplification of the inflammatory response due to generation of more neutrophils and their recruitment and activation. The end-stage disease is determined by damage to bronchial walls by neutrophil-derived reactive oxygen species (ROS) and proteases [5].

These features of the CF lung inflammation have prompted the application of anti-inflammatory treatments, including steroid and non-steroid drugs. However, due to their lack of specificity and significant side effects, new therapeutic interventions counteracting infection and inflammation are being evaluated under cell experiments and clinical trials [6]. Due to the lack of interventional anti-inflammatory drugs in CF, it is necessary to explore and implement novel drugs. In CF, airway epithelial cells are under oxidative stress, which might be due to lack of glutathione (GSH), thiocyanate (SCN−) secretion [7] and ROS accumulation, due to the impaired function of nuclear factor erythroid 2-related factor 2 (Nrf2) [8]. Moreover, retention of F508del-CFTR in the endoplasmic reticulum (ER), excessive production of ROS by mitochondria and alteration of ceramide levels lead to NF-κB and MAPK activation, contributing to the initiation and perpetuation of inflammation [9]. More recently, it has been shown that CF cells exhibited a CFTR-independent intrinsic inflammation and oxidative stress associated with an increase in the intracellular content of Cu [10].

Dimethyl fumarate (DMF), a methyl ester of fumaric acid, is known to reduce cytokine and chemokine gene expression, and to increase anti-inflammatory responses [11,12,13]. These interesting findings have led to increased interest for using DMF in auto-immune or inflammatory diseases, including psoriasis, neurodegenerative diseases and asthma [14,15]. In two phase 3 clinical trials, BG-12, an oral formulation of DMF, has been shown to significantly reduce relapse rates and improve neuroradiologic outcomes relative to placebo in patients with relapsing-remitting multiple sclerosis (MS) [16,17]. DMF exerts its anti-oxidant and anti-inflammatory actions by acting as modulator of the Nrf2 pathway as well as NF-κB translocation [14]. Therefore, it is of interest to explore the interesting properties of DMF in the context of the inflammatory response of CF airway epithelial cells. We show, for the first time, that in a CF cellular model, expressing the *F508del* mutation in homozygosity, DMF drastically reduced both basal and stimulated expression of the pro-inflammatory cytokines IL-1β, TNF-α, IL-6, IL-8 and IL17A. Moreover, we show that DMF also exerts anti-oxidant effects in the CF cells subjected to oxidative stress by various stimuli.

## 2. Materials and Methods

### 2.1. Cell Culture

Experiments were performed in two human immortalized bronchial epithelial cells: 16HBE14o- cells expressing endogenous wt-CFTR (HBE) and CFBE41o- cells stably overexpressing F508del-CFTR (CFBE) [18]. HBE and CFBE cells were maintained in MEM (Corning, New York, NY, USA) supplemented with 10% FBS (Euroclone, Milan, Italy), l-glutamine (Euroclone) and penicillin/streptomycin (Euroclone) at 37 °C, with 5% CO_2_ as previously described [19]. Puromycin (2 µg/mL, Sigma-Aldrich, Milan, Italy) was used as positive selection for CFBE cells.

### 2.2. Cytotoxicity Assay

HBE and CFBE cells (3 × 10^4^ cells/well) were seeded in 96-well plates and exposed to complete medium either in the absence or the presence of various concentrations of (DMF, Sigma-Aldrich; between 1 and 100 μM) for 24 h. Cell viability was evaluated by MTT (3-(4,5-dimethylthiazol-2-yl)-2,5 diphenyl tetrazolium bromide), as previously described [20]. The cell viability was calculated as follows: % viability = (Optical density (OD) of treated cell − OD of blank)/(OD of untreated control − OD of blank) × 100), considering untreated cells as 100%. Cells treated with 1% Triton X-100 were used as positive control.

### 2.3. RNA Extraction and Quantification (qRT-PCR)

Cells were co-treated for 4 h at 37 °C with PBS, 10 µg/mL LPS (Sigma-Aldrich) or 30 ng/mL IL-1β + 30 ng/mL TNF-α or 30 ng/mL IL-1β + 100 µM H_2_O_2_ +/− 50 µM DMF. In another set of experiments, cells were pre-treated with 10 µg/mL LPS for 1 h and then treated with 50 µM DMF for further 4 h. Then, HBE and CFBE were lysed and RNA was extracted according to the manufacturer’s protocol (Qiagen Mini Kit, Hilden, Germany) as previously described [21]. Then, cDNA synthesis was performed while using reverse transcriptase (iSCRIPT cDNA synthesis kit, Biorad, Hercules, CA, USA). Quantitative real-time PCR was performed using EvaGreen fluorophore (Ssofast EvaGreen, Biorad, Hercules, CA, USA). The primers used for amplification were: IL-1β forward: 5′- TTACAGTGGCAATGAGGATGAC-3′; IL-1β revers: 5′- TGTAGTGGTGGTCGGAGATTC-3′, IL-8 forward: 5′-GACCACACTGCGCCAACA-3′; IL-8 reverse: 5′-GCTCTCTTCCATCAGAAAGTTACATAATTT-3, TNF forward: 5′GGACCTCTCTCTAATCAGCCCTC-3′; TNF reverse: 5′-TCGAGAAGATGATCTGACTGCC-3′; IL-6 forward: 5′-CGGTACATCCTCGACGGC-3′; IL-6 reverse: 5′-CTTGTTACATGTCTCCTTTCTCAGG-3′; IL-17A forward 5′-CTACAACCGATCCACCTCACCTTG-3′; IL-17A reverse 5′-GGTAGTCCACGTTCCCATCAGC-3′; GAPDH forward: 5′-CAAGAGCACAAG AGGAAGAGAG-3′, GADPH reverse: 5′-CTACATGGCAACTGTGAGGAG-3′. Fold induction was calculated from the unstimulated controls cells and GAPDH was used to correct for expression.

### 2.4. IL-1β and TNF-α Secretion

Released IL-1β and TNF-α were determined using ELISA kit (R&D Systems, Minneapolis, MI, USA) in supernatants collected from cells stimulated by 10 µg/mL LPS +/− 50 µM DMF for 24 h at 37 °C.

### 2.5. CFTR Channel Function in CFBE Cells

CFBE cells were grown at 37 °C for five days post-confluence submerged on 96-well clear bottom culture plates (Costar, New York, NY, USA) as previously described [22]. Cells were treated with 0.1% DMSO or 3 µM VX-661 + 3 µM VX-445 +/− 10 µg/mL LPS for 24 h. In parallel, 50 μM DMF either alone or in the presence of 3 µM VX-661 + 3 µM VX-445 for 24 h were tested. Then, cells were loaded with blue membrane potential dye and dissolved in a chloride-free buffer for 30 min at 37 °C [23]. The plate was then read in a fluorescence plate reader (FilterMax F5, Molecular Devices, San Jose, CA, USA) at 37 °C. After 5 min baseline, F508del-CFTR was stimulated using 10 µM forskolin (FSK, Sigma-Aldrich) and 1 µM VX-770. After 10 min, CFTR inhibitor (CFTRinh-172, 10 µM, Selleck Chemicals, Houston, TX, USA) was added to deactivate CFTR. The peak changes in fluorescence to CFTR agonists were normalized relative to the baseline fluorescence [24,25].

### 2.6. ROS Measurement

Cells (1 × 10^5^ cells/well) were seeded in a 96-well plate and cultured for 48 h. Then, the cells were co-treated with 10 µg/mL LPS (Sigma) or 30 ng/mL IL-1β + 30 ng/mL TNF-α (R&D Systems, Minneapolis, MI, USA) or 30 ng/mL IL-1β + 100 µM H_2_O_2_ (R&D Systems, Minneapolis, MI, USA) +/− 50 µM DMF for 24 h. The cells were washed with PBS and changed to serum-free media and incubated with 10 µM o5-(and 6)-chloromethyl-2′,7′-dichlorodihydrofluorescein diacetate acetyl ester (H2DCFDA, Invitrogen, Waltham, MA, USA) for 30 min. The conversion of H2DCFDA to fluorescent DCF was measured using a plate reader (FilterMax F5) at 37 °C (excitation: 485 nm; emission 535 nm) [10,26].

### 2.7. Statistical Analysis

All the data are represented as mean with standard deviation. GraphPad 8.0 software (San Diego, CA, USA) was used for all statistical analysis. The paired two-tailed *t*-test or one-way ANOVA were conducted as appropriate with a significance level of *p* < 0.05. Data with multiple comparison were assessed using Turkey’s multiple comparison test with α = 0.05.

## 3. Results

### 3.1. DMF Decreases Cytokine Expression and Secretion in Airway Epithelial Cells

DMF was assessed for its toxicity in regards to both HBE and CFBE cell lines. A 24 h incubation with various concentrations slightly reduced HBE viability, albeit not significantly, whereas CFBE viability did not vary as compared with untreated controls (Figure S1).

**Figure 1:**
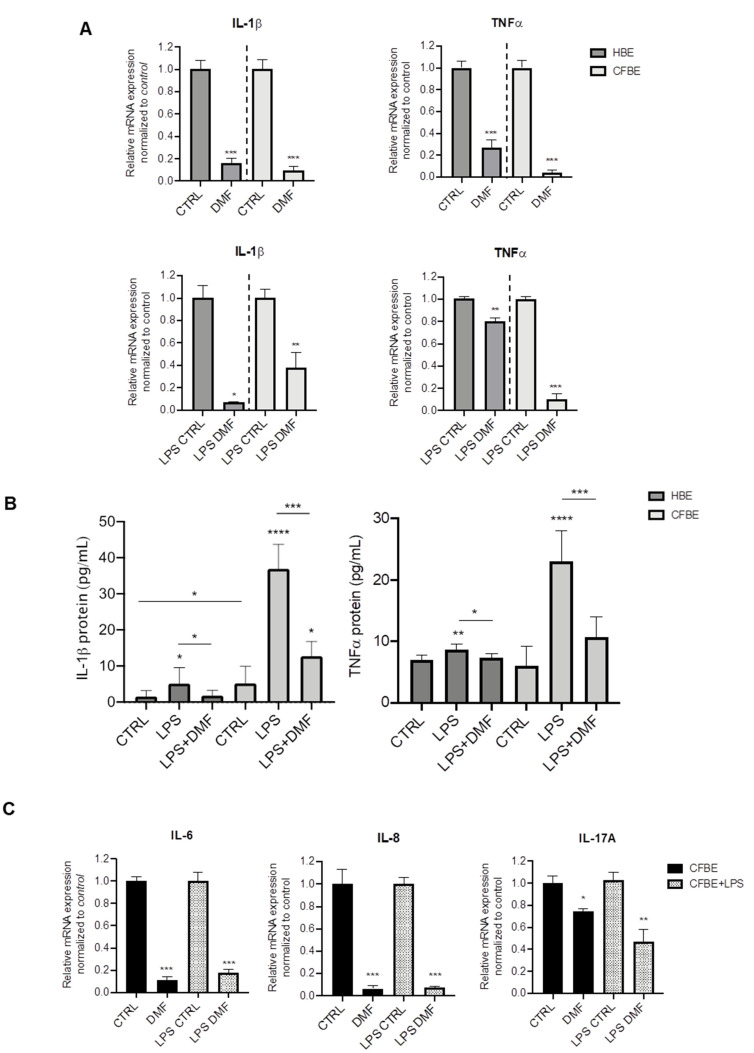
Co-treatment with DMF reduces the expression of IL-1β and TNF-α stimulated by LPS in HBE and CFBE cells. (**A**) Cells were incubated with 0.1% DMSO (CTRL) or 50 µM DMF +/− 10 µg/mL of LPS for 4 h. Total RNA was extracted and qRT-PCR was performed in order to quantify IL-1β and TNF-α mRNA normalized to untreated control. Data represent the mean ± SD (*n* = 4). (**B**) IL-1β and TNF-α secretion after stimulation with 0.1% DMSO (CTRL) or 50 µM DMF +/− 10 µg/mL of LPS for 24 h. Data represent the mean ± SD (*n* = 6–8). (**C**) Cells were incubated with 0.1% DMSO or 50 µM DMF +/− 10 µg/mL of LPS for 4 h. Total RNA was extracted and qRT-PCR was performed in order to quantify IL-6, IL-8 and IL-17A mRNA normalized to untreated control. In (**A**) and (**C**), results are shown as fold induction where CTRL is equal to 1. Data represent the mean ± SD (*n* = 4). * *p* < 0.05; ** *p* < 0.01; *** *p* < 0.001; **** *p* < 0.0001.

Both basal and induced mRNA expression of the pro-inflammatory cytokines IL-1β and TNF-α were assessed by RT-PCR. We first tested the effect of DMF on basal cytokine expression in both cell lines. On the basis of the cytotoxicity test, DMF was used at 50 μM, a concentration that also significantly downregulated inflammatory mediators in many cell types [27,28,29]. As shown in Figure 1A, DMF treatment abated basal expression of IL-1β and TNF-α by ~80% or more in HBE and CFBE cells. For stimulation, we employed LPS from *P. aeruginosa*, one of the mostly used microbial components as a surrogate for whole bacteria. As shown in Figure S2, LPS increased the expression of IL-1β and TNF-α in both HBE and CFBE cells, as indicated by the ΔC_T_ data. To study the effect of DMF on LPS-stimulated cytokine expression, cells were co-treated with DMF and LPS. The co-treatment reduced IL-1β expression in HBE by more than 80%, while this decrease was lower (~60%) in CFBE cells (Figure 1A). On the other hand, TNF-α was significantly inhibited by DMF co-treatment by 80% in CFBE cells and by 20% in HBE cells (Figure 1A).

To validate the mRNA expression studies at the protein level, cytokines were also studied in the conditioned medium (CM) by ELISA assays. Interestingly, IL-1β levels were significantly higher in CFBE CM than in HBE CM either at basal state and after LPS stimulation (Figure 1B). DMF co-treatment determined a significant decrease of IL-1β levels in both cell lines. TNF-α levels were similar in both cell lines and increased with LPS stimulation (Figure 1B), although this effect was lower in HBE cells, as for IL-1β. DMF significantly reduced TNF-α levels after LPS stimulation.

Thus, DMF is capable of suppressing IL-1β and TNF- expression and secretion in wt- and F508del-cells. Since our goal was to investigate the anti-inflammatory effects of DMF in CF cells, the following experiments were performed only in CFBE cells.

To further explore the anti-inflammatory effects of DMF, other cytokines relevant to CF were tested, i.e., IL-6, IL-8, and IL-17A. Figure 1C shows that basal and LPS-stimulated cytokine expression was abated by DMF regarding IL-6 and IL-8. Interestingly, DMF decreased basal and LPS-stimulated IL-17A by only 30% and 60%, respectively.

**Figure 2:**
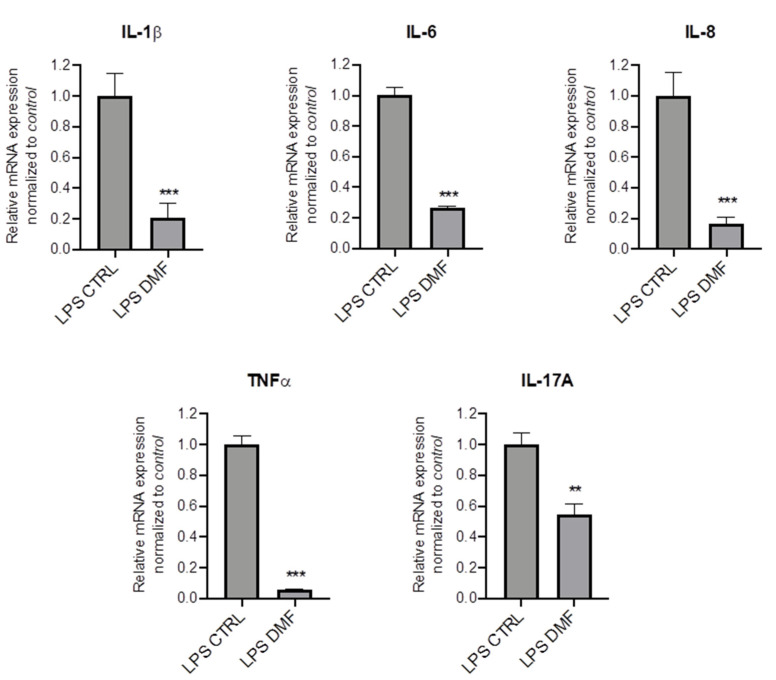
DMF reduces the expression of cytokines stimulated by LPS in CFBE cells. Cells were incubated with 10 µg/mL of LPS for 1 h and afterwards with 0.1% DMSO (CTRL) or 50 µM DMF. Total RNA was extracted and qRT-PCR was performed in order to quantify IL-1β and TNF-α, IL-6, IL-8 and IL-17A mRNA normalized to untreated control. Results are shown as fold induction where CTRL is equal to 1. Data represent the mean ± SD (*n* = 4). ** *p* < 0.01; *** *p* < 0.001.

To investigate whether DMF may be pro-active also in the presence of inflammation, we mimicked the inflammatory milieu of CF airways by pre-treating CFBE cells with LPS for 1 h and then with DMF for 4 h. Results show that DMF significantly reduced mRNA expression levels of different cytokines as compared to cells stimulated only by LPS (Figure 2).

Overall, these results strongly indicate that DMF inhibits a wide range of cytokines associated with CF airway inflammation in a CF cell line, including cells which are already inflamed.

### 3.2. DMF Restores LPS-Inhibited CFTR Activity

**Figure 3:**
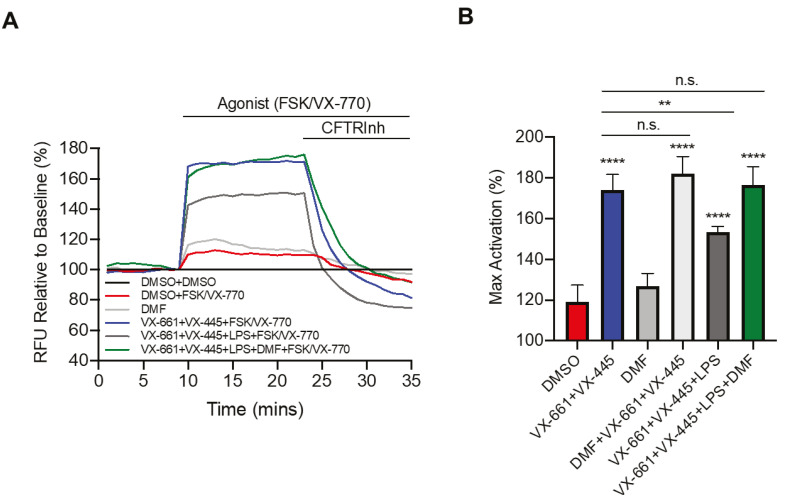
DMF restores VX-661 + VX-445 rescued F508del-CFTR function in CFBE cells. (**A**) Representative traces of F508del-CFTR channel activity using membrane depolarized assay. CFBE cells were treated with 0.1% DMSO, 50 μM DMF, 3 µM VX-661 + 3 µM VX-445, 3 µM VX-661 + 3 µM VX-445 + 50 μM DMF +/− 10 µg/mL of LPS for 24 h. (**B**) Bar graphs show the mean (±SD) of maximal activation of CFTR in different treatment conditions after stimulation by 10 µM Forskolin (FSK) + 1 µM VX-770 (*n* = 4). ** *p* < 0.01; **** *p* < 0.0001.

To further investigate the effect of DMF in the CF context and LPS stimulation, we studied the CFTR activity by the FLIPR assay [30]. Recently, the triple combination VX-661 (tezacaftor) + VX-445 (elexacaftor) + VX-770 (ivacaftor) was approved by the FDA (as Trikafta™) for patients bearing the *F508del* mutation at least on one allele [31,32]. Therefore, we first tested the effect of LPS from *P. aeruginosa* on Trikafta™ treatment. As shown in Figure 3A, pretreatment with VX-661 + VX-445 resulted in a significant improvement in VX-770 potentiated channel activity. Moreover, LPS treatment decreased Trikafta™-mediated F508del-CFTR function in CFBE cells. Interestingly, we found that incubation with DMF restored the CFTR activity to that of non-LPS treated CFBE cells (Figure 3A,B). On the other hand, DMF did not exert any effect on CFTR activity, either alone or in the presence of VX-661 + VX-445, as compared with untreated cells and cells treated with VX-661 + VX-445, respectively (Figure 3B).

### 3.3. DMF Inhibition of Cytokine Expression Is Active on Pro-Inflammatory Stimuli

**Figure 4:**
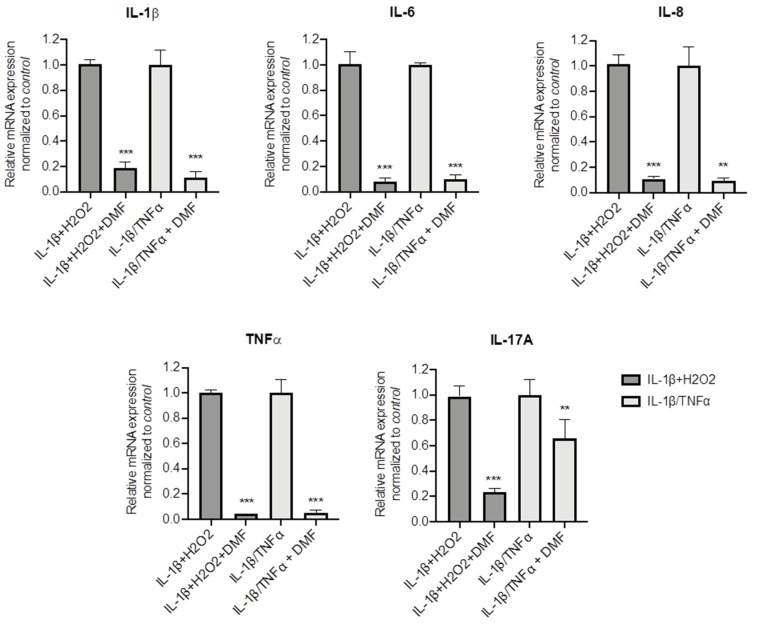
Co-treatment with DMF reduced the pro-inflammatory cytokines expression stimulated by IL-1β + H_2_O_2_ and IL-1β + TNF-α in CFBE cells. Cells were incubated with 0.1% DMSO or 50 µM DMF +/− 30 ng/mL IL-1β + 100 µM H_2_O_2_ or 30 ng/mL IL-1β + 30 ng/mL TNF-α for 4 h. The qRT-PCR was performed in order to quantify IL-1β, IL-6, IL-8, TNF-α and IL-17A mRNA normalized to untreated control. Results are shown as fold induction where CTRL is equal to 1. Data represent the mean ± SD (*n* = 4). ** *p* < 0.01; *** *p* < 0.001.

To better understand whether DMF is capable to dampen inflammation in CF cells, other pro-inflammatory stimuli were used, namely IL-1β + H_2_O_2_ and IL-1β + TNF-α. Both stimuli significantly increased IL-1β, TNF-α, IL-6, IL-8 and IL17A mRNA levels (Figure S3). Figure 4 shows that DMF co-treatment was able to abate cytokine expression under basal conditions and with both stimuli, in regard to IL-1β, IL-6, IL-8 and TNF-α. Notably, while IL-17A mRNA levels were decreased by 80% after IL-1β + H_2_O_2_ stimulation, a lower effect (−40%) was obtained with IL-1β+TNF-α.

### 3.4. DMF Dampens Oxidative Stress in CF Cells

**Figure 5:**
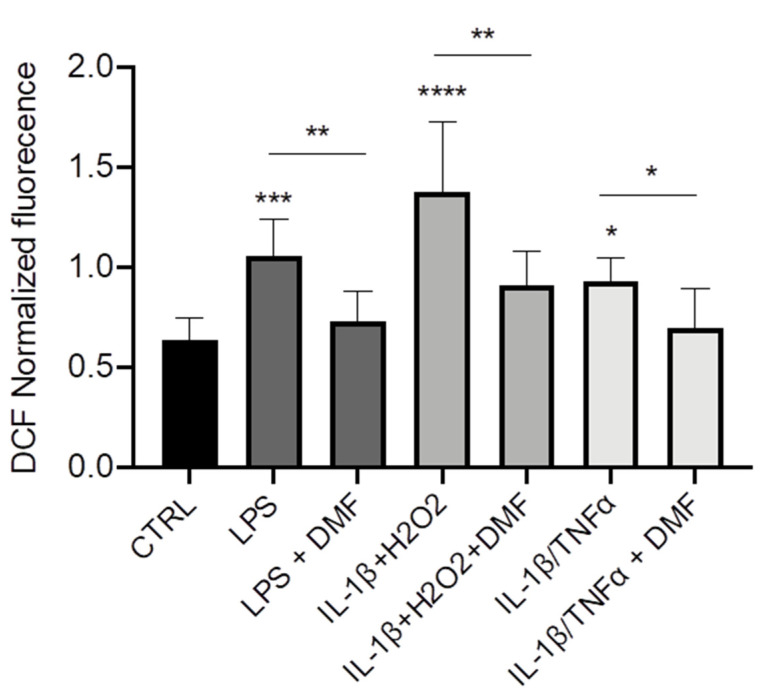
Co-treatment with DMF reduced the reactive oxygen species (ROS) production in CFBE. Measurement of ROS concentration in CFBE stimulated by 10 µg/mL of LPS; 30 ng/mL IL-1β + 100 µM H_2_O_2_ or 30 ng/mL IL-1β + 30 ng/mL TNF-α in presence or absence of 50 µM DMF for 24 h (*n* = 8). * *p* < 0.05; ** *p* < 0.01; *** *p* < 0.001; **** *p* < 0.0001.

Finally, we wanted to understand whether DMF could operate as anti-inflammatory agent via decrease of ROS production. Using a ROS-sensitive fluorescent probe (H2DCFDA), we observed that the intracellular levels of ROS were higher in CFBE stimulated with LPS, IL-1β + H_2_O_2_, and IL-1β + TNF-α as compared with unstimulated cells (Figure 5). DMF co-treatment determined a reduction of ROS levels with all the stimuli and brought them close to those of control cells.

## 4. Discussion

The therapeutic relevance of fumaric acid esters (FAEs), where DMF represents the most pharmacologically effective molecule, has been highlighted in various chronic inflammatory and autoimmune diseases, such as neurodegenerative diseases [14], psoriasis [33,34] and asthma [15]. DMF is known to activate the Nrf2 pathway and to modulate cytokine production via NF-κB and the ERK1/2 and p38 MAPK pathways in many immune cell types [35,36]. Upon exposure to oxidants or xenobiotics, Nrf2 translocates to the nucleus and binds to the antioxidant responsive element (ARE) in the promoter region of genes encoding enzymes involved in the antioxidant response, such as glutathione-S-transferase and heme-oxygenase-1 (HO-1) [37]. Interestingly, Nrf2 exerts its anti-inflammatory actions by inhibiting NF-κB, an oxidant-sensitive transcription factor, due to the increased intracellular GSH levels and GSH-dependent enzymes favoring a reducing environment [38]. However, a continuous pathological stimulus can bring a decline in Nrf2 activity and a persistent increase in NF-κB activity and consequently to chronic inflammation.

In pre-clinical animal models of MS and oxidative stress damage to the brain, DMF has been shown to have beneficial effects on neurodegeneration and toxic oxidative stress, which appear to be mediated predominately through activation of Nrf2-antioxidant response pathway [39,40]. More recently, it has been shown that DMF protected human neuroblastoma cells stimulated with amyloid-beta from ROS-induced damage by activating manganese superoxide dismutase (MnSOD) and HO-1 [41]. BG-12 play a role in dampening inflammatory reactions in psoriasis and MS by inducing tolerogenic dendritic cells [42] and by selectively preventing the nuclear entry of activated NF-κB [12]. Furthermore, DMF inhibited NF-κB and downregulated NF-κB-dependent mediators in psoriasis-relevant cells, i.e., IL-8 and IL-20 in keratinocytes [43] and intercellular adhesion molecule in dermal fibroblasts [44].

At the respiratory level, in primary human asthmatic and nonasthmatic airway smooth muscles, DMF inhibited NF-κB activation and TNF-α-induced eotaxin, RANTES, and IL-6, in addition to PDGF-BB-induced IL-6 expression [13]. In a murine model of experimental asthma, DMF treatment significantly reduced allergen-induced airway inflammation, mucus cell metaplasia and airway hyperactivity, by interfering with the migration of lung dendritic cells to draining mediastinal lymph nodes [45]. DMF have recently been shown to suppress the inflammatory response (studied as *IFNB1*, *CXCL10*, and *CCL5* mRNA levels) to SARS-CoV2 in Calu3 cells, while increasing the expression of the Nrf2-inducible gene HO-1 (*HMOX-1*) [46]. However, to the best of our knowledge, no studies on DMF have been conducted on airway epithelial cells but also on CF.

In this paper, we provide evidence, for the first time, that DMF acts as an anti-inflammatory and anti-oxidant agent in airway epithelial cells expressing the *F508del* mutation. DMF is not effective only under basal but also in stimulated conditions, suggesting its possible role as a therapeutic molecule in an already harshly inflamed CF lung. Indeed, DMF was pro-active as an anti-inflammatory agent either as co-treatment and, even more importantly, as therapy, i.e., when cells were already inflamed. We used three different inflammatory stimuli, such as LPS from *P. aeruginosa* [47,48,49], mimicking the infection, and IL-1β/H_2_O_2_ [50] and IL-1β/TNF-α [51], resembling the inflammatory milieu. Since DMF inhibited both oxidative stress and cytokine secretion, it is tempting to speculate that the anti-oxidant properties of DMF may determine a reduction in the pro-inflammatory potential of CF airway epithelial cells. As shown earlier, CFBE cells can secrete a wealth of cytokines, among which IL-1β, TNF-α, IL-17A, IL-17F and IL-17E are linked to the oxidative stress [10]. In keeping with these data, we confirm that CFBE cells secrete these cytokines and show that DMF inhibits their increased secretion following different stimuli, implying that DMF acts by dampening the oxidative stress-mediated surge of epithelial cytokines. A less inhibitory effect was obtained with IL-17A, a cytokine involved in the amplification of neutrophil activation in the context of anti-bacterial response [52]. Notably, it must be highlighted that IL-17 has been found at elevated levels in chronic lesions in the brain of MS patients [53] and is involved in the MS pathogenesis [54].

Thus, our data generated in CFBE cells indicate that DMF seems to act primarily on the first phases of lung inflammation, dependent on pro-inflammatory cytokines such as IL-1β, TNF-α, and IL-6, and neutrophil chemokines, such as IL-8, all found at high levels in the CF lung secretions.

It has been recently found that LPS-induced CFTR dysfunction in airway epithelial cells is mediated by an early oxidative stress [55]. Indeed, LPS decreased open probability of CFTR and reduced the number of active channels without a concomitant decrease in surface levels of CFTR, which was not due to a cAMP/PKA-dependent effect. Instead, co-incubation with GSH eliminated the impact on post-translational carbonylation of CFTR and reduced CFTR function. In agreement with these data, we show herein that LPS decreased CFTR channel activity as a function of its activation by CFTR modulators such as VX-661 and VX-445, and that DMF recovered CFTR activity. Although we did not study Nrf2 activation following DMF treatment, it is tempting to speculate that DMF operated through an anti-oxidant mechanism. Interestingly, these data indicate that the combination of CFTR modulators and DMF may have a synergistic effect in rescuing CFTR activity while also being anti-inflammatory.

One limitation of our work is that we used continuous cell lines which do not easily translate into meaningful clinically relevant results as compared with the gold standard in CF drug discovery, primary airway epithelial cells [56], especially when they are grown at air–liquid interface (ALI). ALI cultures of primary epithelial cells are closely related to respiratory physiology, since they recapitulate the pseudostratified mucociliary epithelial structure of airways [57]. Thus, our future plan will consider using primary ALI cultures to verify DMF anti-inflammatory and anti-oxidant activities.

In summary, DMF, a pro-drug already used in psoriasis and multiple sclerosis as an anti-oxidant and anti-inflammatory agent, is active in airway epithelial cells bearing the *F508del* mutation. It has two effects: (1) the dampening of the inflammatory potential and (2) the recovery of the CFTR function. Both aspects should be confirmed in more complex models of CF airway epithelium. Nevertheless, this study highlights the relevance of DMF in alleviating CFTR deficiency and inflammation in CF lung disease.

## Data Availability

The data presented in this study are available on request from the corresponding author.

## Supplementary Materials

The following are available online at https://www.mdpi.com/article/10.3390/cells10082132/s1, Figure S1: Cytotoxicity of DMF; Figure S2: LPS-induced mRNA expression levels of primary inflammatory cytokines in HBE and CFBE cells; Figure S3: Analysis of cytokine mRNA expression in CFBE cells after different stimuli.
