# Supplementary material for: Anti-Inflammatory and Anti-Oxidant Effect of Dimethyl Fumarate in Cystic Fibrosis Bronchial Epithelial Cells
Source: Cells. 2021 Aug 19;10(8):2132. doi: 10.3390/cells10082132 (PMC8391758; doi:10.3390/cells10082132)
Supplement: Supplementary file 1 [file cells-10-02132-s001.zip › cells-1289759-supplementary.pdf]

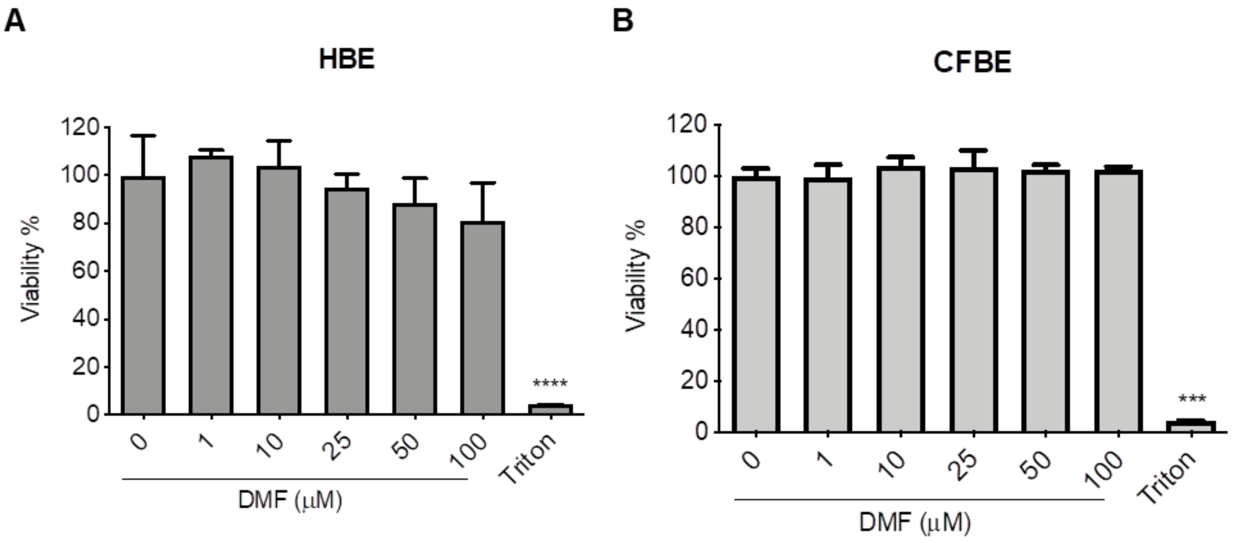

Figure S1. Cytotoxicity of DMF. HBE (**A**) and CFBE (**B**) cells were challenged with DMF for 24 h at the indicated concentrations (in mM). Cells were then assayed for vitality by the MTT assay. Untreated cells represented 100% of viability, whereas 1% Triton X-100 (Triton) was used as positive control. Data represent the mean ± SD (n = 4). ***$p < 0.001$; ****$p < 0.0001$ *vs*. untreated.

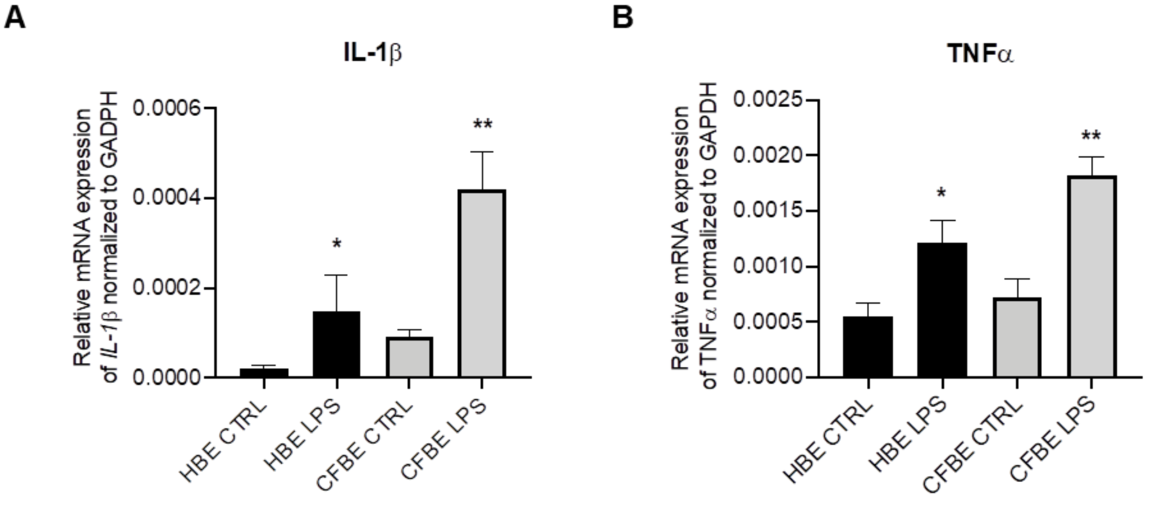

Figure S2. LPS-induced mRNA expression levels of primary inflammatory cytokines in HBE and CFBE cells. Cells were incubated with PBS or 10 μg/mL LPS for 4 h. Total RNA was extracted and qRT-PCR was performed in order to quantify IL-1b (**A**) and TNF-a (**B**) mRNA normalized to GADPH. Results are shown as DC$_T$ values. Data represent the mean ± SD (n = 8). *$p < 0.05$; **$p < 0.01$.

FIGURE S3

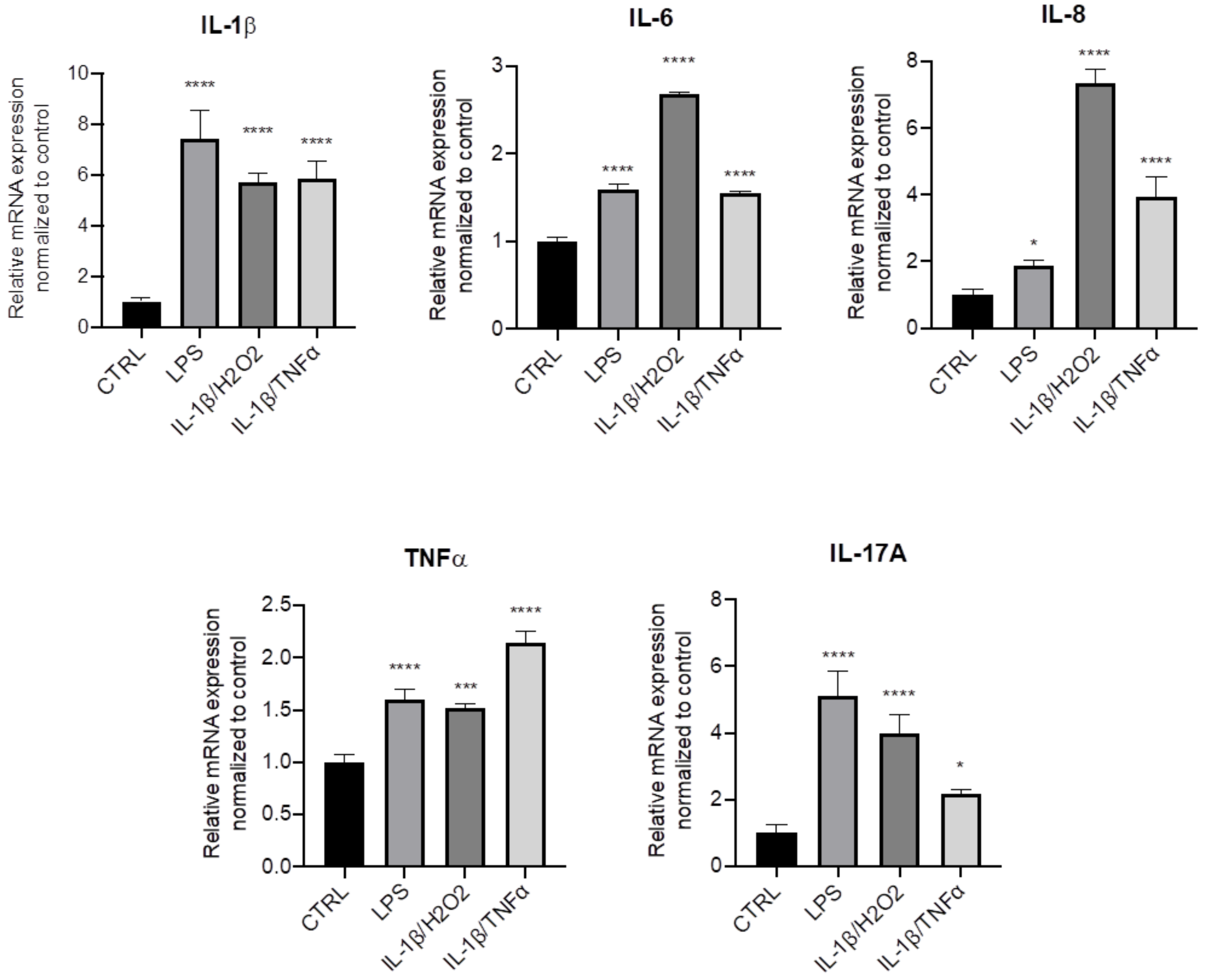

Figure S3. Analysis of cytokines expression in CFBE cells after different stimuli. CFBE cells were incubated with PBS (CTRL), 10 μg/mL of LPS, 30 ng/mL IL-1b+ 100 μM H_2O_2 or 30 ng/mL IL-1b+30 ng/mL TNF-a for 4 h. The qRT-PCR was performed in order to quantify IL-1b, IL-6, IL-8, IL-17A and TNF-a mRNA normalized to untreated control. Results are shown as fold induction where CTRL is equal to 1. Data represent the mean ± SD (*n* = 4). **p* <0.05; ***p* < 0.01; ****p* < 0.001; *****p* < 0.0001.
